# The Influence of Snow Properties on Speed and Gait Choice in the Svalbard Rock Ptarmigan (*Lagopus muta hyperborea*)

**DOI:** 10.1093/iob/obab021

**Published:** 2021-08-14

**Authors:** A Mármol-Guijarro, R Nudds, L Folkow, W Sellers, P Falkingham, J Codd

**Affiliations:** School of Biological Sciences, Faculty of Biology, Medicine and Health, University of Manchester, Oxford Rd, Manchester M139PL, UK; School of Biological Sciences, Faculty of Biology, Medicine and Health, University of Manchester, Oxford Rd, Manchester M139PL, UK; Department of Arctic and Marine Biology, University of Tromso, Hansine Hansens veg 18, Tromso, 9007, Norway; School of Earth and Environmental Sciences, University of Manchester, Oxford Rd, Manchester M139PL, UK; School of Biological and Environmental Sciences, Liverpool John Moores University, Byrom St, Liverpool L33AF, UK; School of Biological Sciences, Faculty of Biology, Medicine and Health, University of Manchester, Oxford Rd, Manchester M139PL, UK

## Abstract

Substrate supportiveness is linked to the metabolic cost of locomotion, as it influences the depth to which the foot of a moving animal will sink. As track depth increases, animals typically reduce their speed to minimize any potential energetic imbalance. Here, we examine how self-selected speed in the Svalbard rock ptarmigan is affected by snow supportiveness and subsequent footprint depth measured using thin-blade penetrometry and 3D photogrammetry, respectively. Our findings indicate that snow supportiveness and footprint depth are poor predictors of speed (*r*^2^ = 0.149) and stride length (*r*^2^ = 0.106). The ptarmigan in our study rarely sunk to depths beyond the intertarsal joint, regardless of the speed, suggesting that at this relatively shallow depth any increased cost is manageable. 3D reconstructions also indicate that the ptarmigan may exploit the compressive nature of snow to generate thrust during stance, as a trend toward greater foot rotations in deeper footprints was found. It remains unclear whether the Svalbard ptarmigan are deliberately avoiding unsupportive snowy substrates. However, if they do, these results would be consistent with the idea that animals should choose routes that minimize energy costs of locomotion.

## Resumen

La firmeza del sustrato se asocial al costo metabólico de la locomoción ya que influencia cuán profundo las extremidades de un animal se hunden al moverse. A medida hundimiento aumenta, usualmente los animales reducen su velocidad para minimizar potenciales desbalances energéticos. En este estudio examinamos cómo la velocidad de la perdiz de la roca de Svalbard es afectada por la firmeza del sustrato y la profundidad de hundimiento de sus patas, usando penetrometría y fotogrametría 3D, respectivamente. Nuestros resultados indican que la firmeza de la nieve y la profundidad de hundimiento de las patas no son buenos predictores de la velocidad (*r*^2^ = 0.149) y de la longitud de la zancada (*r*^2^ = 0.106). La profundidad de las huellas de las perdices de nuestro estudio rara vez sobrepasó la altura de la articulación intertarsal, independientemente de la velocidad de locomoción, sugiriendo que a profundidades relativamente menores los costos energéticos son manejables. Las reconstrucciones 3D también indican que las perdices podrían aprovechar la naturaleza compresiva de la nieve para generar suficiente empuje durante la fase de soporte, ya que se encontró una tendencia hacia mayores rotaciones de la pata en huellas más profundas. Es incierto si las perdices de Svalbard deliberadamente evitan áreas con nieve más blanda. Sin embargo, si lo hacen, estos resultados serían consistentes con la idea de que los animales deberían seleccionar rutas que minimizan los gastos energéticos en locomoción.

## Introduction

Animals traverse complex environments with heterogeneous terrain, where obstacles and variations in substrate, such as ground compliance and roughness, are commonplace. Under such conditions, animals adjust the way they move to maintain stability, maneuverability, and grip to prevent falls and injures (Wilson et al. 1991; Daley et al. 2006; Clark and Higham 2011; Birn-Jeffery and Daley 2012; Li et al. 2012). Animals may also modulate the speed they move at and the route taken, in relation to the energy landscape (Alexander 2000; Shepard et al. 2013). Snow is a common substrate across the temperate and circumpolar regions of the planet. It permanently covers up to 10% of the Earth's surface, and during winter in the northern hemisphere, may extend over 60% of the available surface (Hornberger and Winter 2009). Snow is a fascinating and variable substrate to consider in relation to animal locomotion. When fresh and dry, snow behaves as a fluidizing granular material (Nicot 2004; Hagenmuller et al. 2014); however, temperature increases, rainfall, gravity, and external loadings over time can all lead to changes in the mechanical properties of the snow, creating a spectrum of snow types ranging from very soft, dry snow to crusted hardened layers, to slippery icy layers (Bruland et al. 2004; Nicot 2004). Variability in snow properties can occur rapidly over large and small scales both temporally and spatially.

Changes in snow properties ultimately impact the fitness of the animals that must move over this substrate, as it influences access to food (Hansen et al. 2013; Descamps et al. 2017), reproductive success (Hansen et al. 2013; Descamps et al. 2017), and the metabolic cost of locomotion (Heinonen et al. 1959; Ramaswamy et al. 1966; Fancy and White 1987; Crête and Larivière 2003). In terms of animal movement, any increase in the metabolic cost of locomotion on snow is significant, particularly in areas where snow cover is abundant and there would appear to be a selective advantage for animals opting for specific behavioral strategies to minimize the increase in cost (Shepard et al. 2013). For example, artiodactyls, including the mule deer (*Cervus americanus*) and moose (*Alces alces*), self-select snow pathways where foot sinking depth does not exceed 66% of the height to the chest (Kelsall 1969). Similarly, coyotes (*Canis latrans*) and gray wolves (*Canis lupus*) are known to exploit human-made compressed snow paths to travel more efficiently (Crête and Larivière 2003; Droghini and Boutin 2018b). Many species, including humans, will self-select slower speeds ($U$), relative to when moving over firm ground, to mitigate the increased energetic costs of moving on deep snow with a natural “firm-ground” pace (Ramaswamy et al. 1966; Parker et al. 1984; Crête and Larivière 2003).

Specific anatomical adaptations have also evolved to mitigate the energetic cost increase caused by snow, such as relatively longer limbs and larger foot areas. Relatively longer foot edge lengths may also be advantageous, although empirical evidence of this has only been obtained on natural granular media other than snow (Falkingham et al. 2010). Having a “snowshoe” foot is an effective adaptation for moving over unsupportive snow. For example, the relatively large feet of moose facilitate movement in areas where snow depth exceeds 70 cm, while deer, with their relatively narrow feet, do not move on snow where they might sink deeper than 40 cm because locomotion is severely impeded (Kelsall 1969). A similar relationship is found in lynx (*Lynx canadensis*), which inhabit places with deeper snow when compared with the coyote (*C. latrans*), with their relatively small feet (Murray and Boutin 1991). The snowshoe foot acts as a paddle pushing against the snow for propulsion (Li et al. 2012), evenly distributing the pressure applied to the substrate. Such process may be reflected in the amount of foot subsurface rotation required to transverse through compliant media (Turner et al. 2020). Notably, in other species including the Adélie penguins (*Pygoscelis adeliae*) (Wilson et al. 1991) and the Nearctic river otter (*Lontra canadensis*) (Sadie and Thomas 2005), a common adaptation for moving over snow is switching to a “toboggan” gait, to spread their body weight more evenly when the snow is nonsupportive and deep.

The Svalbard rock ptarmigan (*Lagopus muta hyperborea*) is endemic to Svalbard and is the only bird that permanently lives in this Archipelago. *Lagopus* (from the Latin *lagōpūs,* from the ancient Greek *lagṓ* for “hare” and *poús* “foot”) refers to the feathered foot densely covered in semiplume feathers (Höhn 1977), which in combination with wider and longer claws in winter (Lees et al. 2014) creates a snowshoe that is thought to reduce foot loadings by increasing foot area (Höhn 1977). Ptarmigan face extreme environmental conditions, with temperatures below freezing from September to May and snow cover that persists from October until April–May (Mortensen et al. 1983). Locomotion has been well documented in the ptarmigan and given they regularly commute over snowy ground, they are an ideal species for studying the effects of snow properties on locomotion. Although they can fly, they are predominantly a ground-dwelling bird, with males capable of three terrestrial gaits: walking, grounded running, and aerial running at higher speeds (Nudds et al. 2011). Gaits in the females are restricted to those without an aerial phase (Lees et al. 2012a). Moving over a snowy substrate has also been shown to underpin kinematic differences in grounded running gaits between males and females (Marmol Guijarro et al. 2021). Juvenile ptarmigan rapidly develop adult-like locomotor capacities prior to their first winter (Lees et al. 2012b). Adult Svalbard ptarmigan gain weight as fat reserves for winter, which restricts them to walking and grounded running gaits; however, the males can carry this extra fat—up to 32% of body mass (Mortensen et al. 1983)—at no additional metabolic cost (Lees et al. 2010).

Here, using 3D photogrammetry to measure footprint morphology and measurements of the resistance to penetration of snow, we investigated movements of free-ranging Svalbard rock ptarmigan in the Arctic to examine how snow depth and supportiveness affect track profile, footprint pitch as a proxy of foot subsurface rotation, stride length and the speed of locomotion. We hypothesized that stride length and locomotion speed will decrease as track depth increases on softer, deeper snow. Similarly, we hypothesize that as snow becomes less supportive, footprint pitch will increase. It has been previously proposed that animals may be selecting optimal routes on which to move principally to reduce the associated energetic costs of movement (Alexander 2000); however, there is a general paucity of studies examining animal movement in the wild to test this (Shepard et al. 2013; Marmol-Guijarro et al. 2019). Therefore, we discuss the implications of our results in terms of the energetic budget of wild Svalbard rock ptarmigan.

## Methods

**Fig. 1:**
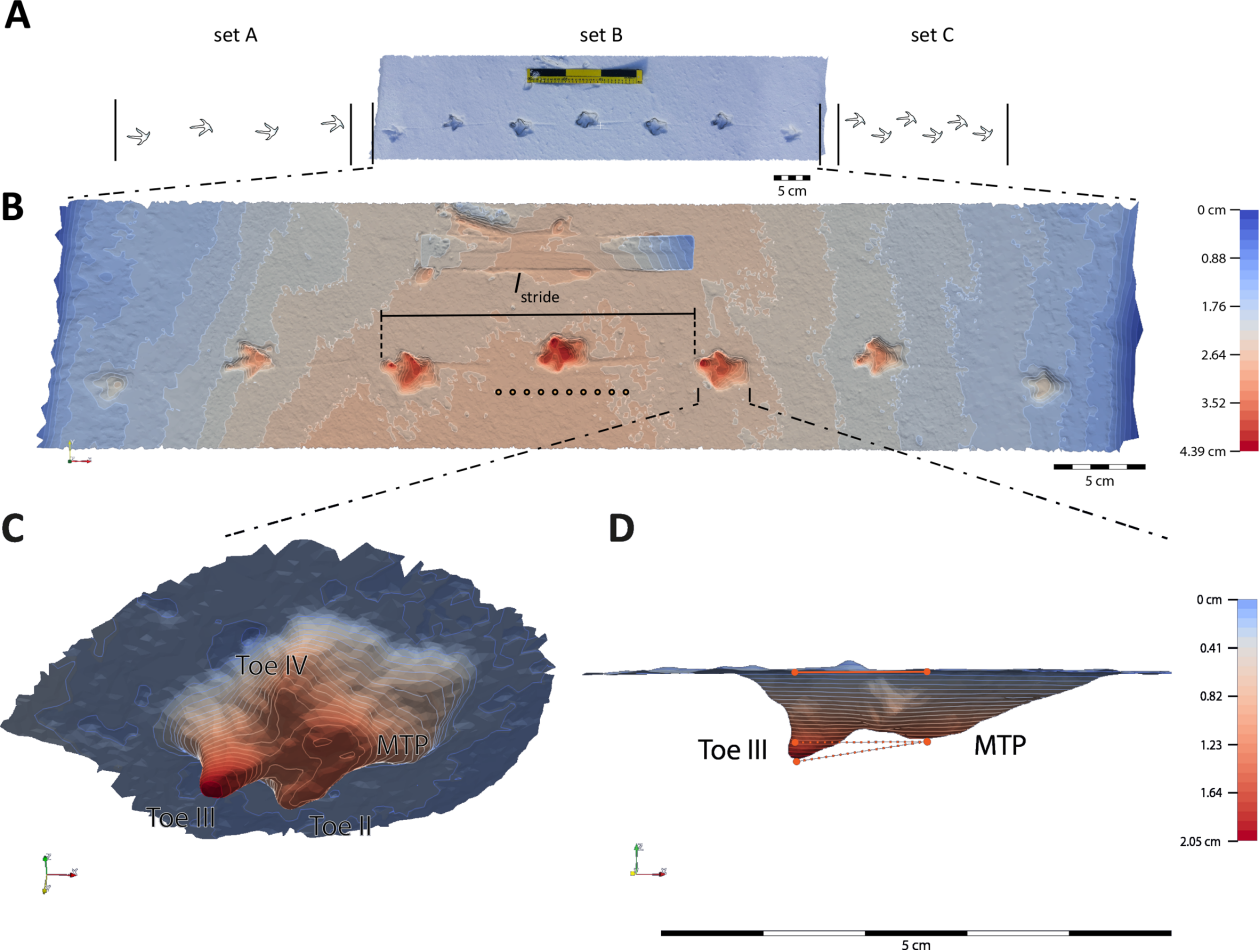
Schematic representation of the footprint analysis in the field. All footprints were taken only from one side. Panel **(A)** represent three sets of continuous footprints where set A and set C were not video recorded, while set B was. A 30 cm scale bar was placed alongside each footprint set and up to 10 force penetration measurements were taken, as a proxy of snow supportiveness (R_snow) (the dots on B). After 3D reconstruction **(B)**, l_stride was measured from the tip of toe III of two continuous footprints. **(C)** D was then estimated as the mean depth at four different footprint landmarks corresponding to toe II, toe III, toe IV, and MTP joint. Finally, the footprint pitch angle was estimated as the angle between toe III-MTP segment and the surface as a proxy of foot subsurface rotation **(D)**.

Data from 14 males were obtained during a field trip to Adventdalen (78°13′18″ N, 15°38′30″ E) and the surrounding side valleys in the Svalbard Archipelago from April 18 to May 3, 2019. During spring, the midnight sun is already present, and the ground is snow covered. Ptarmigan body mass changes seasonally and males are at their summer weights at this time (i.e., no fat reserves are present; Mortensen et al. 1983), thereby being capable of the three terrestrial gaits (Nudds et al. 2011). Males were identified by their secondary sexual characters, including a distinctive red supraorbital comb, a thick black eye-stripe and calls. Birds were recorded moving at self-selected speed ($U$) at 100 frames per second using a SONY^®^ Cyber-Shot RX10 III (SONY^®^ Corporation, Japan) camera on a tripod parallel to the direction of movement at a fixed height and position. Immediately after the bird was out of the shot, a 1 m scale bar was placed in frame (accurate to ±0.01 m), and over the trackway, to allow distance calibration so $U$ could be determined from video recordings using Tracker^®^ v. 5.1.3 (Open Source Physics). A trackway consisted of 1–3 strides, being a stride defined as two subsequent footfalls from the same foot (e.g., left footprint to the next left footprint). Gaits were allocated for a given speed: 0.26 to 0.91 m s^–1^ (walking), 0.92 to 1.48 m s^–1^ (grounded running), and 1.45 to 2.76 m s^–1^ (aerial running), from Marmol-Guijarro et al. (2019). Additional tracks made by the same birds immediately before and/or after the video field of view were also photographed when available (Fig. 1A). For these trackways, stride length (${l_{{\rm{stride}}}}$) was used as a predictor of $U$ for walking and aerial running speeds (Marmol-Guijarro et al. 2020). ${l_{{\rm{stride}}}}$ was measured using ImageJ v. 1.52q (Schneider et al. 2012). Grounded running $U$ was not predicted because of the high error associated with the predictions based on ${l_{{\rm{stride}}}}$ and the lack of certainty of gait identification (Marmol-Guijarro et al. 2020).

### 3D reconstructions and footprint depth analysis

After each video recording, 60 photographs were taken of each trackway from different angles, with a scale bar placed beside the tracks. Then, these photographs were imported into Agisoft Metashape v. 1.5.5 (Agisoft LLC, St. Petersburg, Russia) to generate the 3D models. “Very High Quality” settings were used for image alignment and mesh generation, and texture files were produced. The 3D model resolution depends upon the resolution of the photographs used for reconstruction. A previous study reconstructing 3D models from a cast avian track using 8 megapixel (MP) photographs (*n = *75) reported models with resolutions near 0.3 mm (Falkingham 2012). In our study, we used 60 photographs per track, 20.1 MP each, thereby reconstructing high-quality 3D models of 0.1 mm resolution. The finished 3D models were exported as .OBJ files to CloudCompare v. 2.10.2 (Girardeau-Montaut 2019) to measure the footprint depths ($D$). Prior to the measurements, the footprints of each stride were cropped from the main model to the level of the snow surface. In the majority of trackways where the foot morphology was well preserved, depth measurements from the impressions of Toe II, Toe III, Toe IV and the metatarsophalangeal joint (MTP) were taken (Fig. 1C) and then averaged. In a few deep footprints, the toe regions collapsed after the foot was lifted. In such cases, $D$ was taken from the deepest, most distal portion that remained. The mean footprint depth value ($\bar{D}$) from two successive footprints was taken for analysis of a total of 121 strides with measured $U$ and 186 strides with ${l_{{\rm{stride}}}}$-only obtained from the 3D reconstructions. Previous studies have suggested foot subsurface rotation on granular substrates of varying depths (Li et al. 2012; Turner et al. 2020) and this is an important measure of the functional movements of the foot through any substrate (Turner et al. 2020). To examine this, the footprint pitch angle between the Toe III and MTP segment relative to the surface was measured as a proxy of foot subsurface rotation (Fig. 1D).

### Measuring snow supportiveness

Snow supportiveness (${R_{{\rm{snow}}}}$) was measured using a modified thin-blade force gauge setup (Borstad and McClung 2011) as a proxy for snow hardness to provide a measure of the resistiveness of the snow to penetration. Using an SPK-FMG-008A Chatillon^®^ force probe attached to a Chatillon^®^ DFE2-002 force gauge 10 ± 0.01 N (Ametek Inc., Florida, USA), 10 readings were taken at equidistant points (Fig. 1B) closely beside each track by letting the probe penetrate into the snow up to a fixed depth (1 cm) under its own weight. ${R_{{\rm{snow}}}}$ was then obtained by estimating the peak pressure required to penetrate snow (i.e., force reading divided for the thin-blade area of 0.57 cm^2^) and correcting it by a ptarmigan mean foot area of 5.59 cm^2^ taken from 10 random tracks in this study, which ultimately allowed us to obtained a force measurement closer to what could be observed in the birds.

### Data analysis

All variables were normalized using a log_10_ transformation prior to analyses to ensure that the data met the normality assumptions of the statistical tests used. Quantile–quantile plots were used to confirm this: a normal distribution is achieved if the residuals lie close to the line of best fit of the plot. A linear mixed model (full model, FM) was fitted with log_10_$U$ as the dependent variable, and log_10_${R_{{\rm{snow}}}}$ and log_10_$\bar{D}$ as independent fixed variables including the interaction term (log_10_${R_{{\rm{snow}}}}$ × log_10_$\bar{D}$). The individual contribution of each bird was also included in the statistical model as random effects to account for repeated measures in some individuals. A second linear mixed model using the same independent variables as above was also performed with log_10_${l_{{\rm{stride}}}}$ as the dependent variable. For both dependent variables, the FM models were simplified to assess which statistical model (combination of independent variable) described the data best. Three statistical models in addition to the FM were used. The main effects model (MM) included only fixed effect variables with no interaction term. The snow supportiveness model (RM) refers to a linear mixed model only containing the independent variable log_10_${R_{{\rm{snow}}}}$. The footprint depth model (DM) refers to a linear mixed model only containing the independent variable log_10_$\bar{D}$. The effect of individuals was retained in all the statistical models as a random intercept, and no random slopes were considered as they would render overfitted models due to singularities. To assess which model best fits our data, first, we analyzed the AIC_c_ of all models to estimate the likelihood of the models (*p_i_*) to effectively minimize the AIC_c_. Where AIC_c_ did not discriminate clearly between statistical models—that is, the AIC_c_ of the *i*th model is not significantly different from AIC_c_ of the model with the lowest score (Burnham and Anderson 2004)—the model that explained the largest amount of variation (highest *r*^2^) was considered the best. Thus, a model with the lowest AIC_c_ score or with a significantly comparable AIC_c_ to the lowest AIC_c_ and the largest amount of variation explained was selected as the best model. Although included for completeness (Table 1), the statistical models containing only the intercept were excluded as they would imply that the data are not influenced by any independent variable. To compare the mean subsurface footprint pitch angle between varying depth profiles, we binned all data into 0.5 cm depth ranges and performed a Kruskal–Wallis test followed by a post hoc pairwise Dunn's test to explore the differences between depth profiles. All the statistical analyses were conducted in R v. 3.6.3 (R Core Team 2020), using the lme4 (Bates et al. 2015) and the MuMIn (Bartón 2020) packages to generate the mixed models and to estimate their associated *r*^2^, respectively. We used the Kruskal–Wallis built-in R function for the footprint pitch comparisons, and the Dunn's test function of the FSA (Ogle et al. 2020) package in R.

**Table 1:** Model selection describing the influence of footprint sinking depth (${\bar{D}}$) and snow supportiveness (${R_{{\rm{snow}}}}$) on estimates of speed ($U$) and stride length (${l_{{\rm{stride}}}}$). FM refers to the linear mixed models (LMMs), including both ${R_{{\rm{snow}}}}$ and $\bar{D}$ as independent variables and the interaction term (${R_{{\rm{snow}}}}$ × $\bar{D}$). The MM refers to the LMMs exploring the main effects of ${R_{{\rm{snow}}}}$ and $\bar{D}$ as independent variables without the interaction term. RM and DM are LMMs where ${R_{{\rm{snow}}}}$ and $\bar{D}$ are analyzed independently as single variables. LMMs where neither of the independent variables influenced ${l_{{\rm{stride}}}}$ and $U$ are represented only by the intercept

Model	*K*	AIC_c_	ΔAIC	AIC_wt_	*p_i_*	*r* ^2^
Log_10_$U$						
Intercept	3	−36.44	0	0.412	1	—
**Intercept + log_10_**${{\boldsymbol{R}}_{{{\bf snow}}}}$**× log_10_**${\boldsymbol{\bar{D}}}$**(FM)**	**6**	−**35.83**	**0.613**	**0.303**	**0.960**	**0.149**
Intercept + log_10_$\bar{D}$ (DM)	4	−34.76	1.686	0.177	0.462	0.044
Intercept + log_10_${R_{{\rm{snow}}}}$ (RM)	4	−33.52	2.92	0.096	0.249	0.038
Intercept + log_10_${R_{{\rm{snow}}}}$ + log_10_$\bar{D}$ (MM)	5	−29.33	7.112	0.012	0.033*	0.050
Log_10_${l_{{\rm{stride}}}}$						
Intercept + log_10_$\bar{D}$ (DM)	4	−297.02	0	0.41	1	0.052
Intercept	3	−296.73	0.29	0.355	0.827	—
**Intercept + log_10_**${{\boldsymbol{R}}_{{{\bf snow}}}}$**× log_10_**${\boldsymbol{\bar{D}}}$**(FM)**	**6**	−**295.61**	**1.402**	**0.204**	**0.562**	**0.106**
Intercept + log_10_${R_{{\rm{snow}}}}$ + log_10_$\bar{D}$ (MM)	4	−290.92	6.097	0.019	0.050*	0.053
Intercept + log_10_${R_{{\rm{snow}}}}$ (RM)	4	−289.87	7.15	0.011	0.028*	0.009

*K* is the number of parameters within the model.

*p_i_*-values indicate whether the statistical model's AIC_c_ differs from the model with the lowest AIC_c_.

*r*^2^ corresponds to the explained variance of the fixed effects within the model.

Text in bold indicates the best linear mixed effect model based on the largest *r*^2^ and an AIC_c_ score not significantly different from the model with the lowest AIC_c_.

Ptarmigan individual identity was included as random factor (1|bird id) in all the statistical models to account for repeated measures.

*significant difference.

## Results

The speeds recorded for the Svalbard ptarmigan in this study ranged from 0.20 to 2.39 m s^–1^, the upper limit being 13.4% lower than previous reports in wild males (Marmol-Guijarro et al. 2019). ${l_{{\rm{stride}}}}$ ranged from 0.135 to 0.568 m, which is consistent with what has been reported by Marmol-Guijarro et al. (2019). The Svalbard ptarmigan in this study moved over snow of varying ${R_{{\rm{snow}}}}$, ranging from very soft snow patches resisting peak forces of approximately 1 (N) to hard snow patches resisting up to 67 (N). As expected, the ptarmigan foot sunk deeper into soft snow, and became progressively shallower as ${R_{{\rm{snow}}}}$ increased. $\bar{D}$ ranged from 0.42 cm up to 3.7 cm with only two records exceeding the upper limit by 0.46 and 0.78 cm.

### The effect of snow supportiveness $( {{{\boldsymbol{R}}_{{{\bf snow}}}}} )$ and footprint mean depth $( {{\boldsymbol{\bar{D}}}} )$ on speed $( {\boldsymbol{U}} )$

**Fig. 2:**
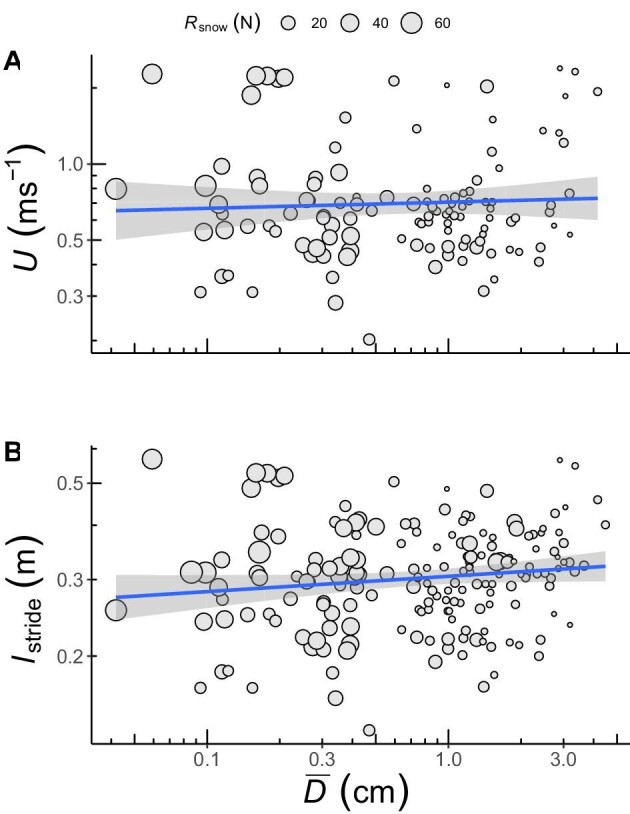
Speed (U) and stride length (l_stride) plotted against footprint depth (D). The circle size varies according to the snow supportiveness (R_snow). The blue solid line represents the line of best fit, while the gray area indicates the 95% confidence interval of the regression line. Although the full models in this study are tridimensional, the plots were fitted as planes for simplicity to the viewer. In panel **(A)**, D and R_snow explained almost 15% of the variation in U, while both variables in panel **(B)** explained little less than 11% of the variation in l_stride.

The FM had the lowest AIC_c_ score and the highest *r*^2^ explaining 14.9% of the total variation in $U$ (Table 1) (Fig. 2A). The effect of ${R_{{\rm{snow}}}}$ on $U$ was not consistent across $\bar{D}$ (*t*_104_ = −3.42, *P *< 0.001). At high values of ${R_{{\rm{snow}}}}$, $\bar{D}$ has no effect on $U$ whereas at low values of ${R_{{\rm{snow}}}}$, $U$ increases linearly with $\bar{D}$. At intermediate values of ${R_{{\rm{snow}}}}$, $U$ again increases with $\bar{D}$, but at a lower incremental rate. The effects of ${R_{{\rm{snow}}}}\ \times \ \bar{D}$ over $U$ are viewed in the interaction plot of Fig. S1. $U$ increased with increasing $\bar{D}$ (*t*_104_ = 3.58, *P *< 0.001), but decreased with increasing ${R_{{\rm{snow}}}}$ (*t*_104_ = −3.44, *P *< 0.001). The intercept (0.864) of the FM differed from zero (*t*_104_ = 3.36, *P *< 0.001). The intercept, however, has little biological meaning here as it suggests that birds move at 7.32 m s^–1^ when ${R_{{\rm{snow}}}}$ and $\bar{D}$ are zero, which is approximately 2.3 times the highest documented $U$ for the Ptarmigan (Lees et al. 2010). Considerably more variation (31.9%) was explained by differences among individuals (random effects) than when only considering the main effects (14.9%).

### The effect of snow supportiveness $( {{{\boldsymbol{R}}_{{{\bf snow}}}}} )$ and footprint mean depth $( {{\boldsymbol{\bar{D}}}} )$ on stride length $( {{{\boldsymbol{l}}_{{{\bf stride}}}}} )$

Again, the FM provided the best fit to the data explaining 10.6% of the variation in ${l_{{\rm{stride}}}}$ and having an AIC_c_ not significantly different from that of the statistical model (DM) with the lowest AIC_c_ (Table 1) (Fig. 2B). The results for ${l_{{\rm{stride}}}}$ were similar to those for $U$. The effect of ${R_{{\rm{snow}}}}$ on ${l_{{\rm{stride}}}}$ was not consistent across $\bar{D}$ (*t*_167_ = −3.41, *P *< 0.001) showing a similar pattern across $\bar{D}$ at different levels of ${R_{{\rm{snow}}}}$ to that found for $U$ (see the interaction plot in Fig. S2). ${l_{{\rm{stride}}}}$ increased with increasing $\bar{D}$ (*t*_167_ = 3.30, *P *< 0.001) and decreased concomitantly with increasing ${R_{{\rm{snow}}}}$ (*t*_167_ = −2.95, *P *< 0.001). Again, the intercept (*t*_167_ = −5.15, *P *< 0.001) by itself lacks any biological relevance. The amount of variation in ${l_{{\rm{stride}}}}$ accounted for by the main effects (10.6%) was once again lower than that accounted for by the random effect, individual (31.4%).

### Footprint subsurface pitch at different footprint mean depths $( {\bar{D}} )$

**Fig. 3:**
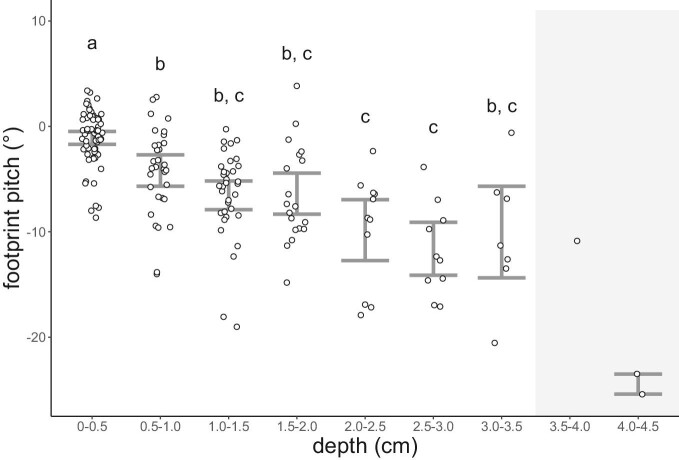
Subsurface rotation of the feet at varying snow depth profiles. Each snow depth profile is binned into 0.5 cm bins from 0 to 4.5 cm. “a,” “b,” and “c” denote distribution similarities in footprint pitch angle based on pairwise Dunn's tests comparisons (Table S3) between all depth profiles. Data points within the gray shaded area were not included in the analyses due to a low sample size.

Our data (Fig. 3 and Table 2) suggest that the ptarmigan foot rotates to a greater extent when pushed deeper into the snow, as the footprint pitch increases with footprint depth (*X*^2^ = 81.2, df = 6, *P *< 0.001). The footprint pitch is mild (1.07° ± 2.61°) when penetrating at the shallowest depths (0–0.5 cm depth) and it is detectably lower than at all deeper profiles. Other than at the shallowest depth profile, and although the pitch angle increases progressively with depth profile, no other depth profile category is statistically discreet from all others (Fig. 3 and Table 2).

**Table 2:** Mean foot subsurface rotation angles at different depth profiles

			95% Confidence intervals	
Depth profile (cm)	*n*	Angle (°)	Lower percentile	Upper percentile
0–0.5	66	1.08 ± 2.62^a^	0.47	1.71
0.5–1.0	32	4.24 ± 4.15^b^	2.85	5.66
1.0–1.5	34	6.41 ± 4.18^b,c^	5.13	7.86
1.5–2.0	19	6.48 ± 4.64^b,c^	4.42	8.45
2.0–2.5	11	9.75 ± 5.27^c^	6.90	12.80
2.5–3.0	10	11.76 ± 4.34^c^	9.09	14.20
3.0–3.5	7	10.24 ± 6.38^b,c^	5.89	14.60
3.5–4.0	1	10.85	—	—
4.0–4.5	2	24.5 ± 1.34	23.5	25.4

^a, b, c^represent distribution similarities in footprint pitch angle between depth profiles determined using Dunn's test pairwise comparisons of each profile.

Data in the shaded areas were not included in the analyses because of their low *n.*

## Discussion

### Effects of snow depth and supportiveness upon speed of locomotion

Our study hypothesized that the ptarmigan travel speed would tend to decrease with decreasing snow supportiveness $( {{R_{{\rm{snow}}}}} )$ and increasing footprint depth $( {\bar{D}} ).$ However, contrary to our hypothesis, $U$ and ${l_{{\rm{stride}}}}$ were only weakly affected by $\bar{D}$ and ${R_{{\rm{snow}}}}$ in our models (*r*^2^ = 0.149 and *r*^2^ = 0.106 for $U$ and ${l_{{\rm{stride}}}}$_,_ respectively). Thus, our data indicate that ptarmigan use similar speed ranges even when moving over snow that offers reduced structural support and that allows the foot to sink to variable depths. This contrasts to the only other data that exist for bipedal locomotion; studies of humans (Heinonen et al. 1959; Ramaswamy et al. 1966) show a reduction in $U$ as sinking depth increases. Reductions in self-selected $U$ are observed in other species, albeit quadrupedal mammals, to keep the energetic cost of moving in deep snow acceptable (Parker et al. 1984; Fancy and White 1987; Crête and Larivière 2003; Droghini and Boutin 2018a). These previous studies also report a positive association between the cost of locomotion and $\bar{D}$. For example, when $\bar{D}$ reaches 30% of the brisket height in elk (*Cervus elaphus nelsoni*), mule deer (*Odocoileus hemionus*), white-tail deer (*Odocoileus virginianus*), and the barren ground caribou (*Rangifer tarandus granti*), the net energy costs of locomotion increase from 12% to 34% (Mattfeld 1974; Parker et al. 1984; Fancy and White 1987). However, if the feet of these species sink to 60% of the brisket height, the energetic costs could span from 111% to 137% of that for a firm substrate, and beyond this point energy costs increase asymptotically (Mattfeld 1974; Parker et al. 1984; Fancy and White 1987). A 100% increase in the energy cost of locomotion has also been reported for coyote-sized dogs moving through 35 cm deep snow (Droghini and Boutin 2018a)—77% of the chest height of a coyote (Murray and Boutin 1991).

In our study, the ptarmigan $\bar{D}$ rarely exceeded 3.71 cm of depth, approximating the mean height to the intertarsal joint of the ptarmigan males (Lees et al. 2012a) and only two incidences during our study (*n *= 182) exceeded this depth (4.15 and 4.48 cm in two different males). These $\bar{D}$ values correspond to 21.4% (25.9% in the case of the extreme value) of the total leg length reported for the Svalbard ptarmigan males elsewhere, although as Lees et al. (2012a) pointed out these values may be underestimated due to the crouched posture of the ptarmigan limb. If it is assumed that similar increases in the cost of locomotion occur in the ptarmigan as in humans—the only other biped for which energetic data on snow of varying depth is available—sinking 25% of the ptarmigan leg length into the snow would imply an increase of more than two-fold in the cost of locomotion compared with firm ground. We did not quantify the energy expenditure of the birds in relation to movement over varying snow depths; however, the fact that the ptarmigan in our study rarely exceeded the intertarsal joint, and that up to this depth there was no evidence that it was affecting $U$, indicates that it is possible that the increase in the energetic cost of locomotion is manageable up to this depth but may increase rapidly if the foot sinks much further. Additional energy savings might also be obtained at bouncing gaits by the ptarmigan as a result of their avian morphology (e.g., horizontally oriented trunk, crouched hindlimb, etc.) (Watson et al. 2011). The snowshoe-like feet of the ptarmigan also contribute to minimize underfoot pressures, preventing them from sinking deeper into the snow, which translates into further energy savings. In humans, for instance, savings of up to 50% of the energy being expended are possible compared with moving without snowshoes (Rogers et al. 1965; Knapik et al. 1997), despite the limitations of carrying a long and heavy device on the feet upon leg kinematics (Browning et al. 2012). In birds generally, and ptarmigan specifically, the avian foot enables a large surface area during stance, but it can also flex into a much smaller area during swing that decreases snow resistance. Hence, it is likely that the energetic costs of locomotion in the ptarmigan are less pronounced than in humans at comparable, relative, footprint depths.

In comparison to the ptarmigan, on the other hand, it appears that human bipeds might be less efficient when walking through deep snow. A study of military personnel, walking at self-selected $U$*,* reported a 120% increase in the metabolic cost of locomotion when subjects sank 30% of the leg length into snow compared with moving over firm ground (Ramaswamy et al. 1966)—a 4–10-fold increase compared with ungulates sinking to an equivalent proportion. The disproportionate increase in the energetic costs of locomotion in humans compared with quadruped mammals may be attributed to the morphological and kinematic differences between both locomotor modes, in particular the greater energetic cost of swinging limbs reported in humans (Pontzer 2007), which may also apply to ground-dwelling birds (Marsh et al. 2004). Bipedal locomotion is only seen obligatorily in humans and birds and occasionally in apes (Alexander 2004), although it is widely accepted that no animal moves in a similar way to humans (Alexander 2004). So, while it is interesting to look for parallels between humans and the ptarmigan, it is worth remembering that there are good reasons why human bipedal locomotion cannot be considered representative. We are not aware of any comparable data from other bird species for direct comparison.

Our study was conducted during early spring. At the onset of winter, from August to October, the Svalbard ptarmigan increase their body mass by 30–50% and this extra mass is maintained until early spring of the following year (Stokkan et al. 1986). This extra mass would translate into higher foot loading being transferred to the snow, which would increase foot sinking depth. Intriguingly, previous work from our group found that despite the additional body mass, the metabolic cost of locomotion is lower in winter (Lees et al. 2010), which, in light of the findings of this current study, we speculate could indicate an adaptation to mitigate the increased costs of the feet sinking at relatively greater depths during the heavier winter snowfalls. However, further study would be required to assess whether there is evidence for any avoidance of very deep soft snow areas, for example using GPS tags together with measurements of the associated metabolic costs (Shepard et al. 2013).

Our models indicate that a large portion of the variance is explained by individuals (repeated measures), which may reflect the necessity to better control the confounding effects of body size and mass. The gold standard in this approach that would enable this to be quantified would be to measure these parameters in each individual bird being recorded; however, this is logistically difficult in a field setting. However, Stokkan and colleagues (1986) found that, at the time of year our current study were conducted, body weight varies by about 30 g in wild caught birds, so this may not be a major issue. Comparable data on body size are not available.

### Footprint morphology

The 3D reconstructions of the footprints suggest greater pitch angles in deeper footprints (Fig. 3, Table 2), indicating greater foot subsurface rotation. Similar results have been seen in guineafowl (Turner et al. 2020) and zebra-tailed lizards (Li et al. 2012) moving on granular media, indicating increased rotation of toe III below the surface with increasing depth (Turner et al. 2020). The ptarmigan may also be taking advantage of the compressive nature of snow by forming a densely packed and supportive snow layer beneath the rotating foot, generating enough ground reaction forces to move forward and leaving a well-defined footprint. A similar phenomenon has been reported in sea turtle hatchlings, where a supportive region of natural sand is created beneath the edge of the flipper after stance and during flipper rotation for thrust (Mazouchova et al. 2010). Irrespective of the substrate, however, it appears that footprint rotation within the substrate may be an additional source of energetic cost.

There is a large source of variation within all the depth profiles included in this study. One possible explanation for this might be related to the snow cover stratification, where layers of snow beneath the surface may vary in their physical properties directly affecting foot rotation and support. For example, a denser layer of snow buried under a layer of fresh new snow at a given depth, or a relatively thin layer of snow above *terra firma*, might prevent subsurface foot rotation—therefore the footprint pitch angle imprinted in snow—as it would provide enough support during the ptarmigan's stance phase. Rocks or other debris may also prevent foot subsurface rotations by keeping one or more toes at shallower depths than the metatarsophalangeal joint if the ptarmigan stand on them. In the same way, footprint pitch may not be accurately estimated in the footprints showing signs of snow collapse. These two factors may obscure a potential relationship between subsurface footprint pitch with ${l_{{\rm{stride}}}}$ and $U$. When plotted together (Fig. S3), a positive trend (differences between ${l_{{\rm{stride}}}}$ ranges are statistically nonsignificant—Table S1) between footprint pitch and ${l_{{\rm{stride}}}}$ is suggested from 0.15 to 0.35 m but no association is evident at longer ${l_{{\rm{stride}}}}$. A similar positive trend is observed between footprint pitch and $U$ up to 1 m s^–1^ (Fig. S4), and again it becomes highly variable at higher speeds (grounded running and aerial running). Footprint pitch angle increases, however, are only detectably different at the lowest speed (Table S2). The lack of an association between footprint pitch angle and higher $U$ and ${l_{{\rm{stride}}}}$, may, in part, be because of low sample sizes at these higher non-walking (grounded and aerial running) speeds.

## Conclusion

Our data indicate that the snowshoe feet of the Svalbard rock ptarmigan can mitigate the potential effects of snow properties on locomotion. In tandem with the seasonal variation in body size and locomotion energy savings during winter reported elsewhere (Stokkan et al. 1986; Lees et al. 2010), these adaptations are key for the ptarmigan to commute in an extreme environment like the one in Svalbard. The birds in this study appear to avoid deep, unsupportive snow patches that would impede locomotion. Our results raise the question as to whether the Svalbard rock ptarmigan are preferentially choosing more favorable routes that do not entail excessive increases in the energetic cost of locomotion. Evidence from other species suggests that this strategy may be widespread as animals seek to maintain their energy balance (Wall et al. 2006). It also questions what feedback mechanisms the birds might potentially be able to use (such as visual cues, through learnt trial and error behavior or real-time kinaesthetic feedback) to identify regions of any substrate that are supportive enough to keep sinking depths reasonable, for example under the intertarsal threshold identified here for the ptarmigan. Further research incorporating real-world quantification of substrate properties and examining their influence on movement and route choice decisions in the context of an energetic landscape would be beneficial.

## Supplementary Material

obab021_Supplemental_File

## Data Availability

Processed data and code are available from figshare digital repository (https://figshare.com/s/709457add73cf386c65a).
