# Supplementary material for: The Influence of Snow Properties on Speed and Gait Choice in the Svalbard Rock Ptarmigan (Lagopus muta hyperborea)
Source: Integr Org Biol. 2021 Aug 14;3(1):obab021. doi: 10.1093/iob/obab021 (PMC8363982; doi:10.1093/iob/obab021)
Supplement: obab021_Supplemental_File [file obab021_supplemental_file.docx]

**Electronic Supplementary Material**


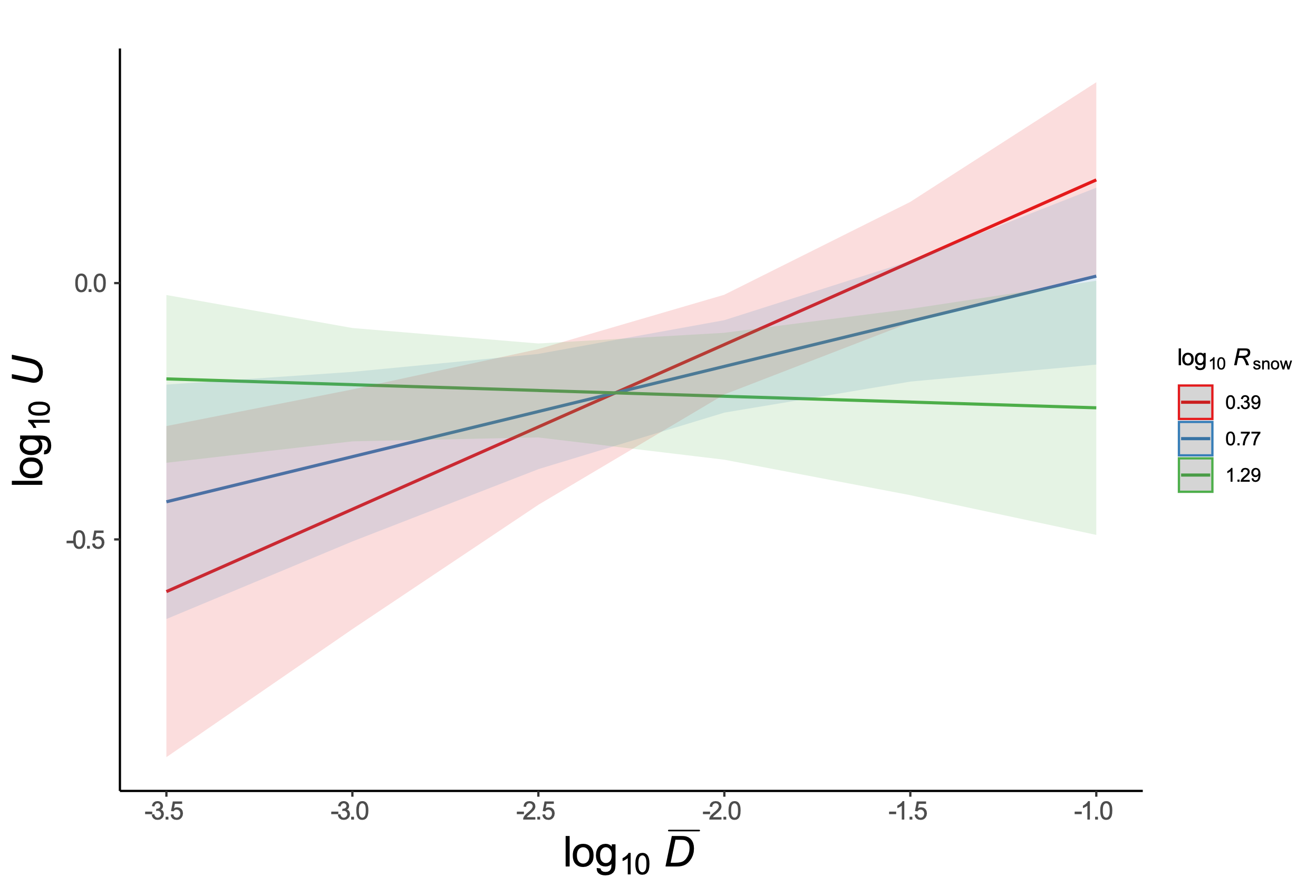


**Figure S1**. Interaction plot for the linear mixed model (full Model-FM) predicting the log_10_ of speed ($U$) from mean footprint depth ($\bar{D}$) and snow supportiveness ($R_{\mathrm{snow}}$). The lines of best fit represent the slope between $U$ and $\bar{D}$ at the first (red), second (blue) and third (green) quartiles of $R_{\mathrm{snow}}$. The shaded areas represent the 95% confidence intervals of the regression lines.


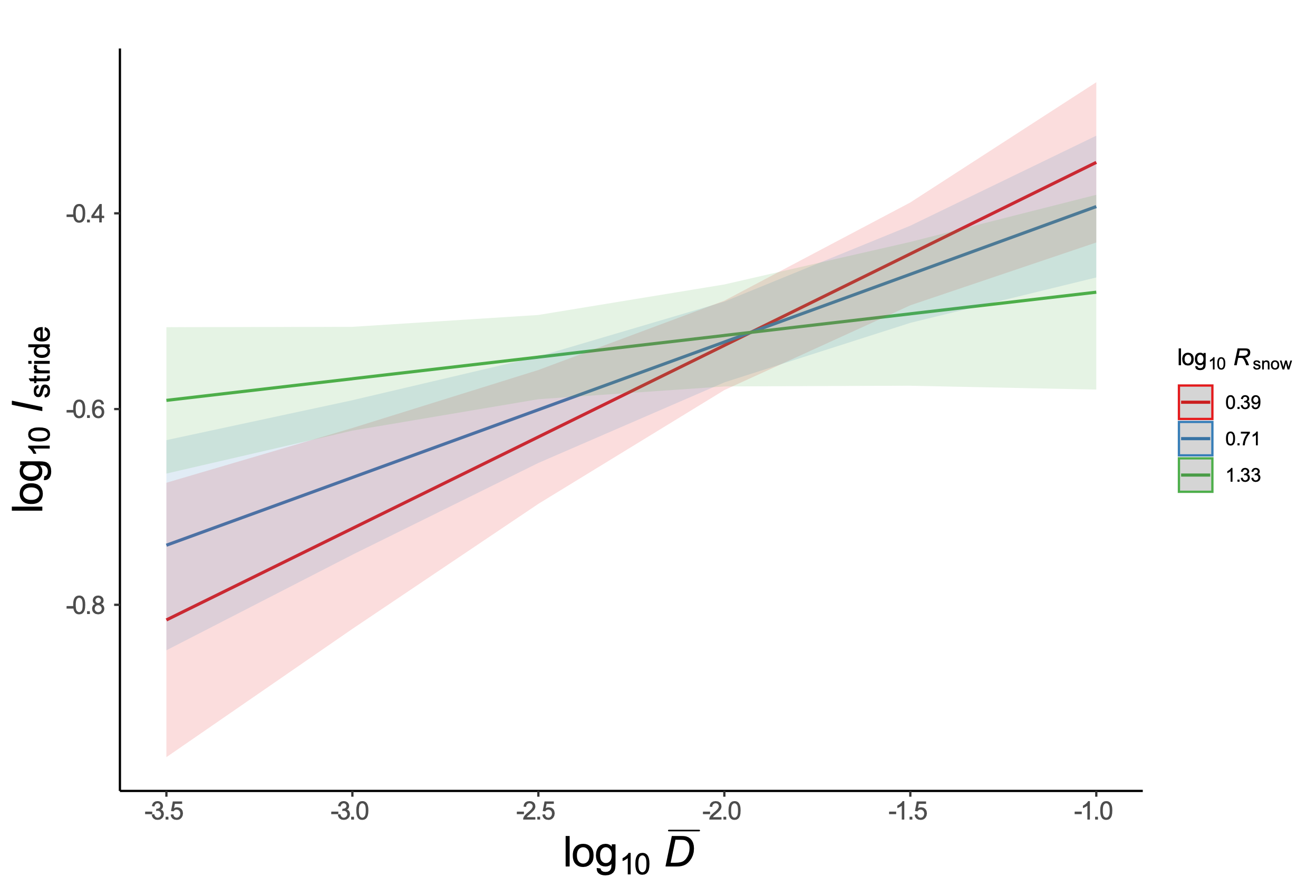


**Figure S2.** Interaction plot for the linear mixed model (full Model-FM) predicting the log_10_ of stride length ($l_{\mathrm{stride}}$) from mean footprint depth ($\bar{D}$) at different values of snow supportiveness ($R_{\mathrm{snow}}$). The lines of best fit represent the slope between $U$ and $\bar{D}$ at the first (red), second (blue) and third (green) quartiles of $R_{\mathrm{snow}}$. The shaded areas represent the 95% confidence intervals of the regression lines.


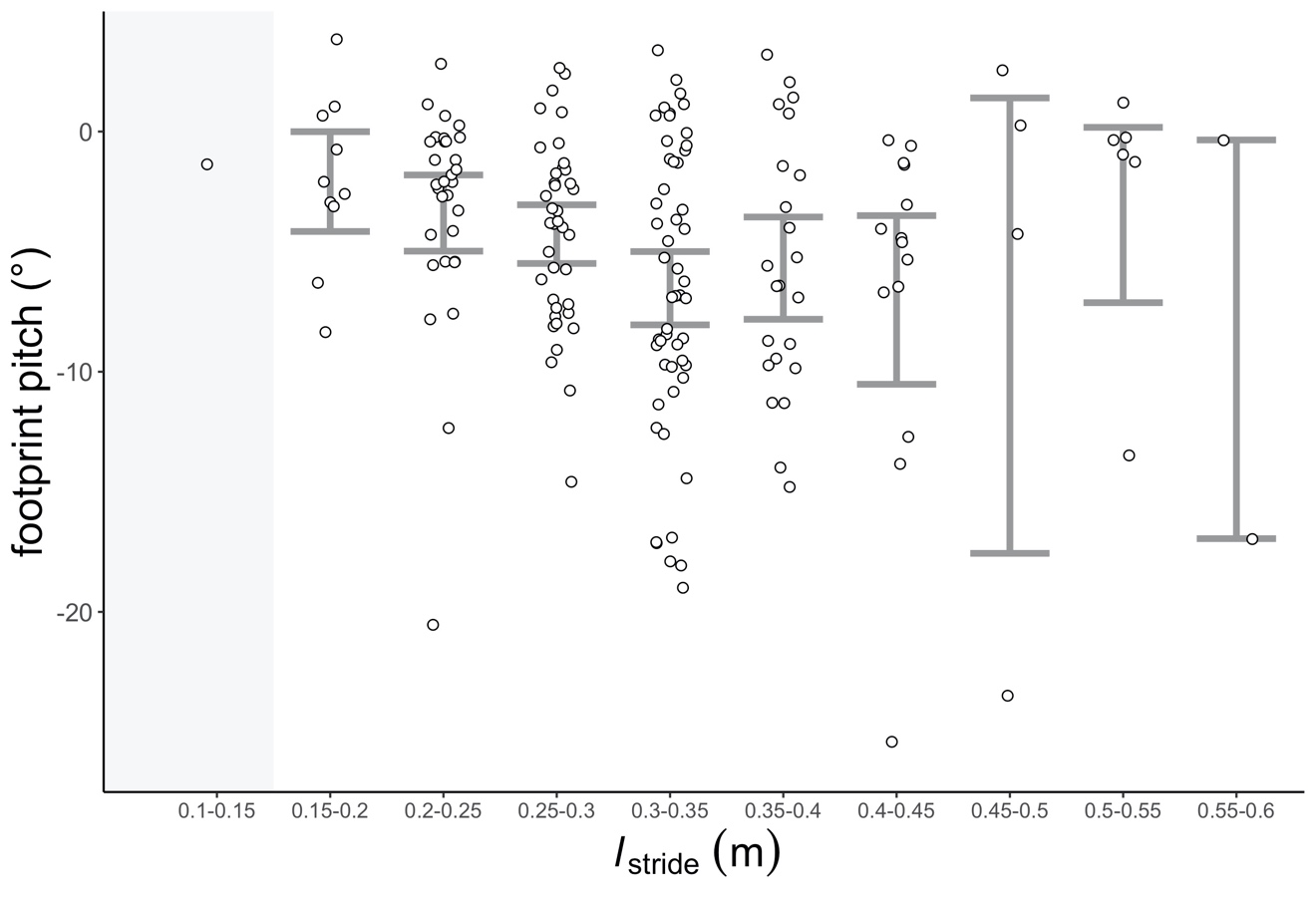


Figure S3. Footprint pitch angle of the feet at different stride lengths (*l*_stride_). *l*_stride_ was binned into 0.05 m bins from 0.10 to 0.60 m. No differences among the *l*_stride_ bins were found based on a pairwise Dunn’s tests. Bars indicate the confidence intervals of the mean for each *l*_stride_ bin. Data points within the shaded area were not included in the analyses due to low sample sizes.


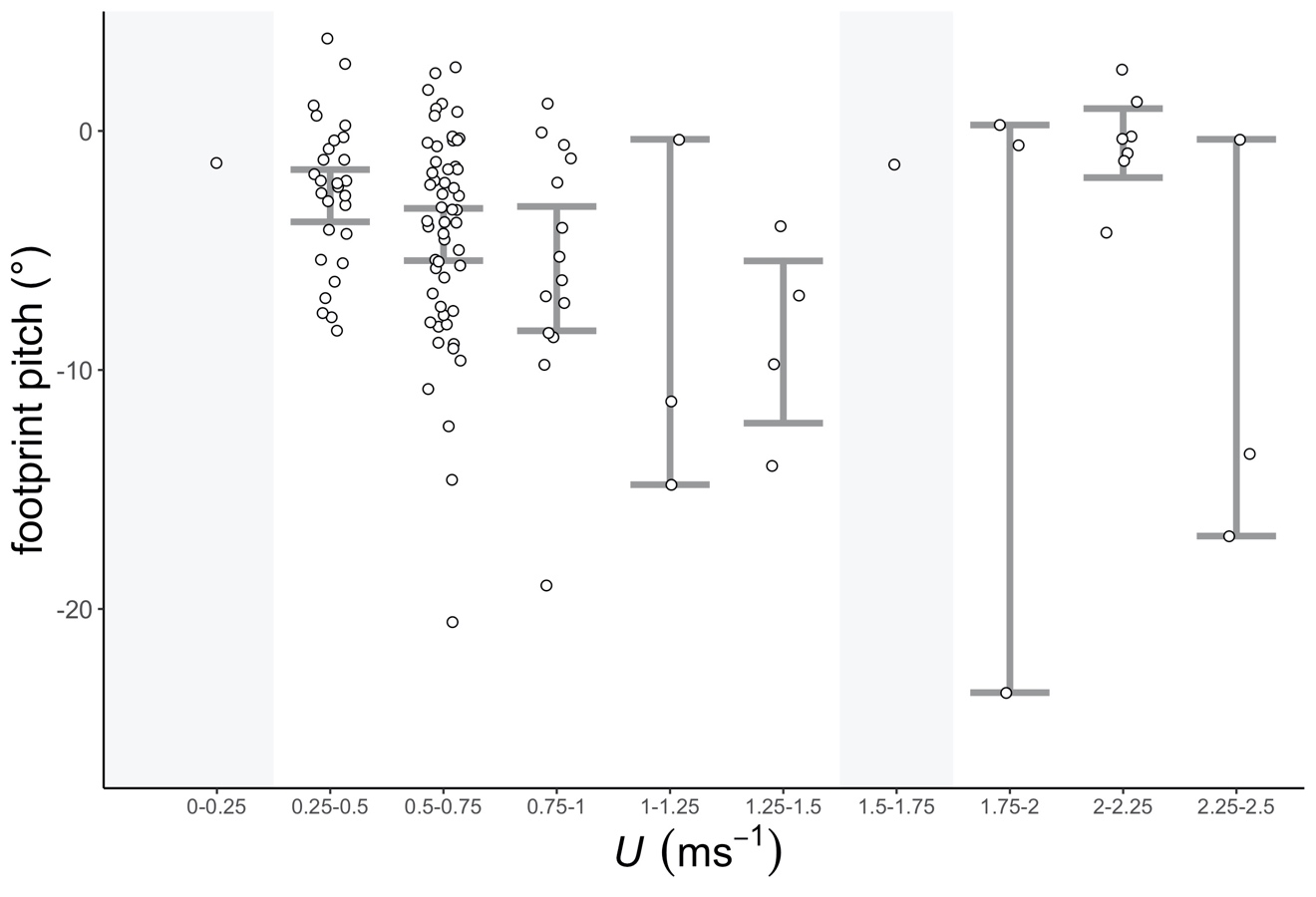


Figure S4. Footprint pitch angle at different speeds (*U*). *U* was binned into 0.25 m bins from 0 to 2.5 ms^-1^. No differences among *U* bins were found based on a pairwise Dunn’s tests. Bars indicate the confidence intervals of the mean for each *U* bin. Data points within the shaded area were not included in the analyses due to low sample sizes.

Table S1. Mean foot subsurface rotation at different ranges of *l*_stride_.

| *l*_stride_ (m) | *n* | Angle (°) | 95% Confidence Intervals | |
| --- | --- | --- | --- | --- |
|  |  |  | Lower percentile | Upper percentile |
| 0.10-0.15 | 1 | 1.35 | - | - |
| 0.15-0.20 | 10 | 2.06 ± 3.55 ^a^ | 0.02 | 4.24 |
| 0.20-0.25 | 31 | 3.19 ± 4.46 ^a^ | 1.78 | 4.85 |
| 0.25-0.30 | 39 | 4.22 ± 3.84 ^a^ | 3.04 | 5.44 |
| 0.30-0.35 | 52 | 6.55 ± 5.98 ^a^ | 4.98 | 8.18 |
| 0.35-0.40 | 23 | 5.67 ± 5.24 ^a^ | 3.55 | 7.77 |
| 0.40-0.45 | 14 | 6.45 ± 6.81 ^a^ | 3.46 | 10.20 |
| 0.45-0.50 | 4 | 6.24 ± 11.85 ^a^ | -1.40 | 17.60 |
| 0.50-0.55 | 6 | 2.52 ± 5.45 ^a^ | -0.19 | 7.06 |
| 0.55-0.60 | 2 | 8.65 ± 11.74 ^a^ | 0.35 | 17.00 |

^a^ indicates distribution similarities in footprint pitch angle bins determined using pairwise comparisons (Dunn’s tests) of each *l*_stride_ profile.

Data in shaded areas were not included in the analyses due to a low *n.*

Table S2. Mean foot subsurface rotation at different ranges of *U*.

| *U* (ms^-1^) | *n* | Angle (°) | 95% Confidence Intervals | |
| --- | --- | --- | --- | --- |
|  |  |  | Lower percentile | Upper percentile |
| 0-0.25 | 1 | 1.35 | - | - |
| 0.25-0.50 | 10 | 2.62 ± 3.12 ^a^ | 1.49 | 3.76 |
| 0.50-0.75 | 31 | 4.26 ± 4.46 ^a^ | 3.09 | 5.46 |
| 0.75-1.00 | 39 | 5.60 ± 5.22 ^a^ | 3.16 | 8.46 |
| 1.00-1.25 | 52 | 8.82 ± 7.54 ^a^ | 0.35 | 14.80 |
| 1.25-1.50 | 23 | 8.66 ± 4.26 ^a^ | 5.44 | 12.20 |
| 1.50-1.75 | 1 | 1.40 | - | - |
| 1.75-2.00 | 4 | 7.95 ± 13.47 ^a^ | -0.25 | 23.50 |
| 2.00-2.25 | 6 | 0.47 ± 2.13 ^a^ | -0.94 | 1.98 |
| 2.25-2.50 | 2 | 10.27 ± 8.76 ^a^ | 0.35 | 17.00 |

^a^ indicates distribution similarities in footprint pitch angle determined by pairwise comparisons (Dunn’s tests) of each *U* profile using the *p-*adjusted values.

Data on shaded areas were not included into analysis due to their low *n.*

**Table S3.** Dunn’s test pairwise comparisons output for foot rotation over different (binned) depth profiles summarised in Figure 2 and table 2.

| Comparison | *Z* | *p* | *p* - adjusted |
| --- | --- | --- | --- |
| 0-0.5 vs. 0.5-1 | -3.49 | 4.82E-04 | 1.69E-03 |
| 0-0.5 vs. 1-1.5 | -4.98 | 6.40E-07 | 4.48E-06 |
| 0.5-1 vs. 1-1.5 | -1.12 | 0.262 | 0.324 |
| 0-0.5 vs. 1.5-2 | -4.38 | 1.19E-05 | 6.26E-05 |
| 0.5-1 vs. 1.5-2 | -1.28 | 0.199 | 0.279 |
| 1-1.5 vs. 1.5-2 | -0.34 | 0.732 | 0.768 |
| 0-0.5 vs. 2-2.5 | -5.13 | 2.83E-07 | 2.98E-06 |
| 0.5-1 vs. 2-2.5 | -2.63 | 0.008 | 0.020 |
| 1-1.5 vs. 2-2.5 | -1.89 | 0.059 | 0.103 |
| 1.5-2 vs. 2-2.5 | -1.48 | 0.140 | 0.210 |
| 0-0.5 vs. 2.5-3 | -5.78 | 7.33E-09 | 1.54E-07 |
| 0.5-1 vs. 2.5-3 | -3.34 | 0.001 | 0.002 |
| 1-1.5 vs. 2.5-3 | -2.64 | 0.008 | 0.022 |
| 1.5-2 vs. 2.5-3 | -2.18 | 0.029 | 0.061 |
| 2-2.5 vs. 2.5-3 | -0.66 | 0.506 | 0.591 |
| 0-0.5 vs. 3-3.5 | -4.11 | 3.88E-05 | 1.63E-04 |
| 0.5-1 vs. 3-3.5 | -2.12 | 0.034 | 0.065 |
| 1-1.5 vs. 3-3.5 | -1.49 | 0.137 | 0.221 |
| 1.5-2 vs. 3-3.5 | -1.18 | 0.238 | 0.313 |
| 2-2.5 vs. 3-3.5 | 0.08 | 0.940 | 0.940 |
| 2.5-3 vs. 3-3.5 | 0.66 | 0.507 | 0.560 |
